# Ocular Manifestations of Alzheimer's Disease in Animal Models

**DOI:** 10.1155/2012/786494

**Published:** 2012-05-15

**Authors:** Miles Parnell, Li Guo, Mohamed Abdi, M. Francesca Cordeiro

**Affiliations:** ^1^Glaucoma & Retinal Neurodegeneration Research Group, Visual Neuroscience, UCL Institute of Ophthalmology, London EC1V 9EL, UK; ^2^Sutton Eye Unit, Epsom and St. Helier NHS Trust, Cotswold Road, Sutton, Surry, London, UK; ^3^St. Georges Healthcare NHS Trust, Blackshaw Road, Tooting, London, UK; ^4^Western Eye Hospital, Imperial College Healthcare Trust, London, UK

## Abstract

Alzheimer's disease (AD) is the most common form of dementia, and the pathological changes of senile plaques (SPs) and neurofibrillary tangles (NFTs) in AD brains are well described. Clinically, a diagnosis remains a postmortem one, hampering both accurate and early diagnosis as well as research into potential new treatments. Visual deficits have long been noted in AD patients, and it is becoming increasingly apparent that histopathological changes already noted in the brain also occur in an extension of the brain; the retina. Due to the optically transparent nature of the eye, it is possible to image the retina at a cellular level noninvasively and thus potentially allow an earlier diagnosis as well as a way of monitoring progression and treatment effects. Transgenic animal models expressing amyloid precursor protein (APP) presenilin (PS) and tau mutations have been used successfully to recapitulate the pathological findings of AD in the brain. This paper will cover the ocular abnormalities that have been detected in these transgenic AD animal models.

## 1. Introduction

Alzheimer's disease (AD) is a form of dementia affecting 26.6 million people worldwide as of 2006; a figure predicted to quadruple by 2050 [1]. It manifests clinically with progressive cognitive impairment that can be divided into a predementia phase and mild, moderate, and severe dementia phases which are increasingly accompanied by noncognitive and neurological disturbances [2]. 

There has been much work investigating the pathogenesis of AD which has resulted in several key findings allowing formation of several hypotheses. 

The most well-regarded theories as to the pathology underlying the degeneration of neurons in the brain are the formation of extracellular senile plaques (SPs) via amyloid-beta (A*β*) deposition [3] and formation of intracellular neurofibrillary tangles (NFTs) via hyperphosphorylation of tau protein [4]. Indeed the presence of SPs and NFTs remain the mandatory pathological findings to make a definitive diagnosis [5], a stipulation that has not changed since they were first described in 1906 by Alois Alzheimer [6].

The exact mechanisms as to how A*β* deposition and NFT formation cause neurotoxicity and neuronal loss remain unclear although several factors have been postulated. These include increased oxidative stress by production of reactive oxygen species by A*β* [7, 8], increased oxidative stress mediated by metal ions within A*β* [9], and interaction of A*β* peptides with the N-methyl-d-aspartate (NMDA) receptor resulting in neurotoxicity [10]. Additional theories of AD pathogenesis include chronic inflammation [11] and reduced synthesis of acetylcholine [12]. The different hypotheses proposed are summarised in Table 1. 

In fact, to go back a step in the pathogenesis of AD, it is also not clear what causes the aggregation of A*β* as amyloid plaques; however, there is much evidence that it involves the dyshomeostasis of metal ions. A*β* is known to precipitate out in the presence of primarily zinc but also copper and iron ions [20] due to a high-affinity metal-binding site [21], and occupation of this binding site has been shown to prevent the formation of A*β* from APP by inhibiting the initial cleavage of APP by *α*-secretase [22]. The relevance to AD is clear in that pathology only seems to occur when A*β* precipitates out and forms plaques. Many studies have demonstrated that A*β* plaques contain high levels of zinc and copper [23, 24]. The potential significance of zinc in the aetiology of A*β* formation and therefore AD is underscored by the fact that the A*β* plaque load experienced by APP transgenic mice is attenuated by crossing with mice that lack a zinc transporter and are thus unable to transport zinc into synaptic vesicles [25]. In the same vein, it is also worth noting that A*β* plaques are concentrated in the most zinc-rich area of the brain, the hippocampus [26], and levels of hippocampal zinc have been shown to be higher in AD brains than age-matched controls [27]. A comprehensive review of AD and metal dyshomeostasis is provided by Barnham and Bush [28].

Much of the insight we have gained with regards A*β* and tau pathology has been obtained from specific genetic mutations that account for a subset of AD (approximately 1% of the disease as a whole) which is inherited in an autosomal dominant manner [29]. There are three genes that have been identified that cause familial AD (FAD). The first FAD mutation was found in the APP gene located on chromosome 21 [30]. So far, twenty different mutations affecting the APP gene causing FAD have been defined [31]. The two other locations of FAD mutations are presenilin 1 (PS1) on chromosome 14 [32, 33] and presenilin 2 (PS2) on chromosome 1 [34, 35]. 

Although much progress has been made in our understanding of AD, this has not as yet been translated into either effective treatments or, crucially, an earlier or more accurate diagnosis.

Currently, a firm diagnosis of AD requires histological analysis of central nervous system (CNS) tissue to find the pathological changes described above and is thus a postmortem one. Making a clinical, premortem diagnosis relies on assessment of cognitive impairment and memory loss and has been reported to be inaccurate in 10–15% of cases [36], presumably due in part to the similarity of symptoms to other diseases such as depression and other forms of dementia. In addition, a clinical diagnosis is less than ideal because, firstly, it is difficult in the early stages to distinguish AD from normal ageing effects and, secondly, the ability to detect cognitive impairment implies a significant amount of damage has occurred already, making possible treatments less likely to be effective. 

These problems have led to significant efforts in identifying biomarkers that could be used to diagnose and monitor AD. There has been some promise in using magnetic resonance imaging (MRI) to determine differential patterns of brain atrophy [37] as well as using positron emission tomography (PET) scanning to detect labelled A*β* plaques [36]. Other studies have shown that AD patients have higher levels of tau protein and lower levels of A*β*42 in their cerebrospinal fluid (CSF) [38–41]. As worthy as these efforts are, they are not yet accurate enough to be useful for diagnosis with, for example, the use of MRI to detect atrophy only able to differentiate from normal subjects in 85% of cases [37, 42, 43], which is not an improvement on clinical diagnosis. There is also the not inconsiderable cost of these techniques to consider, especially in the context of the prevalent nature of AD, as well as the difficulties of compliance in this group of patients. 

A particularly promising strategy is the eye. Although reluctant to quote a cliché that has been adapted by the scientific community from an even more hackneyed phrase, it is true that the eye can be considered a window to the brain. The retina exists as an extension of the CNS, and, thanks to its purpose of receiving light and translating into vision, it is optically transparent. Changes that occur in the retina can be visualised noninvasively and directly with increasingly sophisticated imaging techniques. As impressive as noninvasively detecting labelled A*β* plaques with PET imaging is [36], it is a far cry from the ability to detect changes in single neurons as is now possible in the eye [44–47].

Historically, the visual symptoms that have long been reported in AD patients (see later) have been attributed to neuronal damage to the visual pathways in the brain rather than the retina [48, 49]; however, there is increasing evidence that the specific pathological findings in the brain occur in the retina also, both in AD patients and transgenic AD animal models.

## 2. Ocular Manifestation in Alzheimer's Patients (Table 2)

### 2.1. Visual Deficits in AD

Various different aspects of vision have been reported to be affected in AD since Cogan's findings in 1985 [71]. These include abnormalities in visual acuity, [50, 51], contrast sensitivity [52, 72], colour vision [54, 55], and motion perception [73, 74].

### 2.2. Retinal Abnormalities

 As would be expected, the majority of the changes that have been observed in AD eyes are in the retina. 

Cross-sectional imaging of the retina using optical coherence tomography (OCT) has demonstrated in various studies that AD is associated with thinning of the peripapillary retinal nerve fibre layer (RNFL) [62–64] with the loss occurring superiorly initially [65]. These in vivo findings correspond with the predominantly inferior visual field loss experienced by AD patients [57] and are corroborated by histopathological findings of reduced number of RGCs and axonal degeneration in postmortem AD retinas [75–77]. In vivo imaging has also suggested a correlation of AD severity and reduced thickness of the RNFL (presumably due to loss of RGCs and axonal degeneration) at the macula [63] as well as a decrease in RNFL thickness and neuroretinal rim of the optic nerve head [78].

In vivo Doppler imaging techniques have demonstrated a decrease in retinal blood flow [65] analogous to cerebral blood abnormalities demonstrated in AD [79], although whether this is a primary phenomenon or simply a consequence of a thinned retina is hard to say.

Another noninvasive technique which evaluates retinal function is electrophysiology. Changes in pattern electroretinograms (PERG) and visual evoked potentials (VEPs) have been noted in AD patients [66–68] with specific changes being correlated with RNFL thickness [69].

APP and A*β* immunoreactivity has been detected in an age-dependent manner [80] in the retinas of AD patients, and, even more excitingly (in terms of finding a retinal biomarker for AD), A*β* plaques have been demonstrated as a postmortem finding in the retinas of AD patients [70]. A comprehensive review has been published on this topic elsewhere [81].

### 2.3. Other Ocular Abnormalities

AD patients have been noted to suffer from a particular type of cataract, namely, equatorial supranuclear, with A*β* deposition localised to the opacities [61]. Similar cataracts have been noted in Down's syndrome subjects, further supporting deposition of A*β* in the lens as the cause [82]. This raises the intriguing possibility that one could detect A*β* in the lens as a screening tool for AD although clearly this would require the changes in the lens preceding the symptoms of AD which has not as yet been proven. It would be interesting indeed if an ocular biomarker was found for AD that is not connected to the CNS.

A summary of these visual pathway signs and symptoms that have been described in AD patients is presented in Table 2.

These findings make a compelling case to investigate ocular manifestations of AD further, and, for the remainder of this paper, we shall look at what has been discovered using animal models of AD.

## 3. Animal Models of Alzheimer's Disease

FAD accounts for less than 1% of AD cases [83, 84] and has been shown to be due to the three genes mentioned above (APP, PS1, and PS2) inherited in an autosomal dominant manner. Their relatively small contribution to the AD burden as a whole is inversely proportional to the amount of information we have gleaned from them, both in terms of forming and confirming hypotheses about the pathogenesis, and providing animal models.

The use of animal models in investigating any disease process is important as they provide a way of standardising the disease among subjects and can be experimented on in ways that are simply not possible in human subjects.

Most of the animal models of AD are mice as, firstly, they are mammals and therefore have a similar CNS structure to humans, and, secondly, it is *relatively* easy and inexpensive to produce transgenic strains expressing one or more of the elucidated genes. 

Currently, transgenic mice come in three varieties: single, double, or triple transgenic simply referring to the number of genes they express. The earliest mice models were single transgenic which increased A*β* by increasing APP via a mutant APP gene. Early examples include the PDAPP mice which express an FAD mutation containing a valine residue substitution at position 717 using a platelet-derived growth factor-*β* promoter [85] and the Tg2576 mice which express a different FAD mutation characterised from a Swedish family of FAD sufferers (K670N/M671L) using a hamster prion promoter [86]. Both these lines expressing different FAD-associated mutations and various others that have been designed since [87–89] have shown amyloid deposition and glial activation that increases with age [86, 90] and overall have been successful in mimicking these neuropathological aspects of AD as well as cognitive deficits [88]. 

Although these different models do show significant similarities across different studies, there are differences such as timing of onset of the amyloid plaques [91] which is presumably explained by the different host strains, different promoters, and different specific mutations of each model.

APP mutations account for only a small proportion of FAD [31], and mice expressing FAD mutations of PS1 and PS2 have also been created. Transgenic mice containing either FAD PS1 or PS2 mutations show elevated levels of the relatively amyloidogenic A*β*42; however, these lines do not go on to develop plaques [92, 93], a finding that can be explained by the fact that mice and rats lack two histidines that make up the A*β* metal binding site [20, 94, 95] and highlight further the potential importance of zinc dyshomeostasis mentioned earlier. 

A natural follow on from this is to create double transgenic mice containing both APP and PS1 or PS2 mutations. Various combinations have been created and investigated, and, in general, the addition of a PS1 or PS2 mutant gene to the APP mutant gene accelerates the rate of amyloid deposition and plaque formation [96–99].

A triple transgenic mouse model was created in 2003 containing APP, PS1, as well as tau transgenes [100] which successfully recapitulated the amyloidogenic as well as the NFT features of AD with the mice developing amyloid plaques as well as NFTs.

More recently, rat models using the same principles have been established that show similar rates of amyloid deposition [101–103] and have the theoretical advantage that behavioural studies will be more achievable than in mice.

Other avenues explored in rodent models are over expressing endogenous APP, knockout mice, mutations in beta, gamma, and alpha secretase, ApoE, however, it is beyond the remit of this paper to describe all these in detail and the reader is directed toward a database of AD animal models kept by the Alzheimer research forum at http://www.alzforum.org/res/com/tra and two comprehensive reviews on AD animal models from Spires and Hyman and from Duyckaerts et al. [31, 104].

Worth briefly mentioning are nonrodent models of AD which, while having the obvious disadvantage of being so phylogenetically distinct from humans, have the advantages that they are easier, cheaper, and quicker to perform experiments on. The Drosophila fruit fly contains a homolog of APP [105] and presenilin [106] as does the nematode ceanorhabditis elegans [107, 108]. Overexpression of these endogenous proteins as well as transgenic expression of human mutant APP, PS, and tau genes in these species has certainly contributed to this field however not to the same extent as their rodent counterparts.

The remainder of this paper will focus on what we have learnt specifically from the eyes of these animal models.

## 4. Ocular Manifestations of Alzheimer's Disease in Animal Disease Models

### 4.1. Retinal Changes (Table 3)

#### 4.1.1. Amyloid *β* Deposition

Several studies using transgenic AD mouse models have demonstrated the presence of A*β* in the retina. One study by Liu and colleagues [109] used the previously described single transgenic Tg2576 mouse model which contains the APP double Swedish mutation and shows an age-dependent deposition of extracellular A*β* and amyloid plaques in the cerebellum, hippocampus, and cortex as well as displaying cognitive deficits [86]. In this study, they demonstrated extracellular A*β* immunoreactivity and plaque like formation using four different monoclonal antibodies as well as Congo red staining in Tg2576 retinas. The A*β* deposition occurred predominantly from the ganglion cell layer to the outer nuclear level with plaques even found in the photoreceptor layer and optic nerve head. Another study that utilised the Tg2576 mouse model was less successful however in detecting A*β* deposition [111]. Here, A*β* immunoreactivity was tested using the A*β* monoclonal mouse antibody 1E8 and was only found in the retinal periphery with no plaque like structures detected. Of relevance is the fact that, in the same study, plaque-like structures were found in the cerebral cortex of the same animals using the same antibody. Also interesting to note is that the differences in the studies cannot be explained by a disparity in age of the animals as Liu et al. mice were aged 14 months as were the animals used by Dutescu et al. suggesting, perhaps, a differential sensitivity of the A*β* antibodies used.

Retinal A*β* deposition has also been found in double and triple transgenic mouse models expressing APP and PS mutations. One study used two different strains of APP/PS mice [112]. In the first strain which contained mutant human APP and PS1 genes (Tg2576 × Tg1), they found that extracellular A*β* deposition, as determined by immunoreactivity to a monoclonal mouse A*β* antibody, was present predominantly in the nerve fibre layer and ganglion cell layer in animals aged 27 months but not at the younger age of 7.8 months. In the second strain containing the same APP mutant gene but with a different PS1 gene (APP_swe_/PS1_ΔE9_), there was a similar pattern of A*β* immunoreactivity predominantly in the nerve fibre and ganglion cell layer although these animals were at an intermediate age of 10.5 months. This second strain (APP_swe_/PS1_ΔE9_) has been used in two subsequent studies facilitating a comparison of sorts. In one of these studies by Perez and colleagues [113], A*β* plaques, as determined by thioflavin-S and confirmed with immunostaining, were found from 12 months old but predominantly in the inner and outer plexiform layers with far fewer plaques present in the GCL, INL, and ONL. This fits in with the temporal findings of Ning et al.'s study but not with the localisation. Another study utilising the same mouse model is from Dutescu and colleagues [111]. In this study, moderate A*β* deposition was detected in the GCL, IPL, INL and OPL in 9-month-old mice. Overall these results seem rather inconclusive; however, the lack of A*β* plaques found in Ning and Dutescu's work is potentially explained by the fact that they were looking at an earlier time point than the earliest point at which plaques were detected (12 months) in Perez' study.

One study has further characterised that the relatively amyloidogenic form of A*β*, A*β*42, is deposited in the GCL, INL, and ONL of APP single transgenic and APP/PS1 double transgenic mice [114]. 

Recent studies using a triple transgenic mouse model expressing APP, PS1, and tau mutations have also demonstrated increased A*β* deposition across the retina, particularly in the GCL and the inner segments of photoreceptor [47, 115]. 

A particularly exciting finding is that, in double transgenic mice (APP_swe_/PS1_ΔE9_), retinal A*β* plaques can be stained with curcumin and imaged safely in vivo [70]. In the same study, they demonstrated that the retinal plaques detected ex vivo occurred prior to plaques in the brain. This is hugely relevant as it suggests that retinal changes could potentially be used to make a diagnosis of AD, noninvasively, prior to even the current gold standard of postmortem histological analysis.

A*β* has also been detected in the retinal and choroidal vasculature of animal models in keeping with the corollary in the brain; cerebral amyloid angiopathy [110, 116, 117]. In 27-month-old double transgenic mice (Tg2576 × Tg1), A*β* immunoreactivity was detected in both retinal and choroidal microvasculature which was not present in younger (7.8 months) animals. At an intermediate age (10.5 months) A*β* immunoreactivity was present in the choroidal vasculature only [112]. It should be noted that the intermediate aged animals, although double transgenic for APP and PS1 as with the 7.8- and 27-month old animals, contained different mutations. Another study using Tg2576 single transgenic mice [109] detected A*β* deposition in retinal capillaries of 14-month-old mice. Interestingly, administration of amyloid peptide vaccinations increased this vascular deposition despite decreasing the extracellular plaques which mirrors what occurs in brains of mice models [118] and supports the theory that immunotherapy solubilises A*β* allowing it to drain via the vascular system [119].

#### 4.1.2. APP Immunoreactivity

As one would expected APP has been detected in the retina of the same animals that exhibit A*β* deposition. 

In single transgenic Tg2576 mice which overexpress APP, Liu et al. [109] detected immunoreaction of APP in the GCL and INL of 14-month-old animals. This was corroborated in a separate study using the same animals of a similar age [111]. 

In double transgenic mice (Tg2576 ×  Tg1), APP was likewise detected in the GCL and INL of 27-month-old animals although was not present in younger, 7.8-month-old, mice. Unlike the Tg2576 single transgenic mice, APP was detected to a small degree in the RPE and photoreceptors [112]. In the same study, a different double transgenic strain (APP_swe_/PS1_ΔE9_) exhibited APP immunoreactivity only in the GCL at an intermediate age of 10.5 months. The same animal model in a different study [111] showed moderate APP staining in the IPL and OPL in 9-month-old animals.

APP immunoreactivity has also been detected in the retina of a double transgenic mouse model containing the Swedish APP mutation and a PS1 knock in, although this study did not clarify in which layers this was confined to [114].

#### 4.1.3. Tau Protein

The hyperphosphorylation of the microtubule-associated protein tau and subsequent deposition as neurofilbrillary tangles is associated with various neurodegenerative disorders (collectively called tauopathies) such as progressive supranuclear palsy, frontotemporal dementia, and parkinsonism linked to chromosome 17 and AD [120, 121]. Tau inclusions have been observed in the brains of AD transgenic mice [100, 122, 123] and appear to be a feature of the AD retina as well. In single transgenic Tg2576 mice overexpressing APP, hyperphosphorylated tau was detected using the AT8 antibody adjacent to the A*β* deposition from the GCL through to the ONL [109]. A different mouse model that expresses the human P301S tau transgene and develops tau inclusions throughout the central nervous system [124] is used as a model for tauopathies rather than specifically AD. In this transgenic line, hyperphosphorylated tau was found in the RNFL which progressed to tau inclusions in the GCL with associated deleterious effects on axonal growth [120].

#### 4.1.4. Neuroinflammation

Activated microglia and astrocytes are thought to initiate neuroinflammation in AD and have been shown to be upregulated in the brains of mouse models of AD [125, 126]. Microglial activation in the retina has been shown in mouse models of retinal degeneration [127]. It is then relatively unsurprising that significant upregulation of inflammation has been detected in the retinas of AD mouse models.

In Tg2576 single transgenic mice, there was increased activation of astrocytes and microglia in all layers of the retina compared to wild-type controls as detected using cell-specific markers GFAP for astrocytes and IBA1 for microglia [109]. Immunisation with amyloid peptide vaccinations in the same study led to increased neuroinflammation in the retina in accordance with similar findings in the brains of AD mouse models [128].

In double transgenic mice (APP_swe_/PS1_ΔE9_), microglial activation was significantly higher than in age-matched controls as detected using a macrophage marker F4/80 [113]. The same study used GFAP to look for astrocytic activation and found that there was no measurable upregulation.

In the same double transgenic model aged 10.5 months, another study found that monocyte chemotactic protein (MCP) 1, a relatively nonspecific marker of inflammation was increased in the GCL (the same area as A*β* deposition was occurring) compared with wild-type controls although F4/80 immunoreactivity was not significantly different [112]. A different double transgenic model (Tg2576 × Tg1) in the same study found that, at a younger age, F4/80 and MCP-1 immunoreactivity was significantly less than at a higher age of 27 months leading them to conclude that this was due to progression of AD and that the MCP-1 but not F4/80 immunoreactivity in the intermediate aged mice represented a relatively early stage of inflammation prior to microglial activation. While this may well be true, the lack of a wild-type control at the young and old ages makes it hard to be sure that this is not merely an ageing effect independent of AD.

Another finding that suggests an important role of neuroinflammation in the propagation of AD and indeed other neurodegenerative disorders is a consistent downregulation of complement factor H (CFH) in AD brain [129]. CFH is a cofactor that acts to suppress the alternative complement pathway; hence, low levels of CFH have a proinflammatory effect. One paper evaluated the presence of CFH and A*β*40 and A*β*42 peptides in the brains and retinas of several different transgenic AD mouse models (Tg2576, PSAPP, 3 × Tg-AD, and 5 × FAD) and found that there was a consistent inverse correlation between levels of A*β* and CFH in the retinas of these transgenics [130] suggesting that an environment promoting complement activation is a feature of AD retinas. Interestingly, CFH has also been implicated in the pathogenesis of AMD [131], another neurodegenerative disease affecting the retina suggesting, perhaps, a similar contribution of neuroinflammation to these diseases. Research into AMD has suggested a link between CFH and zinc. Zinc has been shown to cause aggregation of CFH monomers [132] which, combined with the high levels of CFH and zinc [133] that are found in the sub-RPE deposits (drusen) that characterise this disease, suggest a critical role for zinc analogous to the one it is postulated to play in AD.

Unfortunately, to the best of our knowledge, there is no research that has looked at levels of zinc in the retina of AD animal models.

#### 4.1.5. Neuronal Cell Loss

In common with other neurodegenerative diseases, cell death and loss of neurons is an end stage of AD. 

In double transgenic mice (Tg2576 × Tg1), a significant increase in apoptosing cells in the GCL of 27-month-old animals compared with 7.8 month old animals was detected using terminal deoxynucleotidyl transferase mediated dUTP nick end labeling assay (TUNEL) [112]. The same study also found an increase in TUNEL-positive cells in the GCL of a different double transgenic model (APP_swe_/PS1_ΔE9_) compared with age-matched controls.

In single transgenic Tg2576 mice, using retinal thickness as a measure of neuronal loss, a reduced thickness was detected compared with wild-type controls [109]. In addition, administration of amyloid peptide vaccinations attenuated the reduction of retinal thickness in conjunction with a reduction of A*β* deposition in these animals.

Using TUNEL to look at NMDA-induced apoptosis in APP and PS1 single transgenic mice and APP/PS1 double transgenic mice has yielded potential insight into how A*β* may cause retinal degeneration [114]. In this study, APP and APP/PS1 transgenic mice displayed fewer TUNEL-positive cells in the GCL following injection of NMDA than wild-type controls suggesting that deposition of A*β* may prevent activation of NMDA-receptor pathways and mediate retinal dysfunction in AD in this way. In the same study, however, there was no detected difference in RGC number or INL thickness (obviously a relatively crude measure of neuronal loss) between the APP overexpressing single transgenic mice, APP/PS1 double transgenic, and PS1 knockin mice and their wild-type controls. This is clearly at odds with other studies and may represent a difference in the strains used as well as less sensitive methods of counting cells.

A relatively recent development now allows direct visualisation of apoptosing ganglion cells in the retina. Using a fluorophore labelled annexin V protein as a marker of apoptosis and confocal laser scanning ophthalmoscopy to detect the fluorescence, it is possible to image single apoptosing ganglion cells in real time and in vivo [44, 46]. This technique has been refined and used to visualise apoptosing (labelled with annexin V) and necrosing (labelled with propidium iodide (PI)) cells in a triple transgenic mouse model of AD [47]. In this study, the triple transgenic mice displayed increased RGC apoptosis and decreased RGC necrosis compared with wild-type controls.

### 4.2. Other Ocular Changes

For obvious and very sensible reasons, the retina has been the target of most of the research looking at AD in the eye. It has though been established that the lenses of AD patients, as mentioned earlier, contain A*β* aggregates that colocalise with a specific type (equatorial supranuclear) of cataract [61]. This appears to be similarly manifested in AD mouse models with the epithelial cells of the corneas and lenses of single transgenic Tg2576 mice and double transgenic APP/PS1 mice, being immunopositive for APP and A*β* [111].

## 5. Conclusion

The idea of using the retina as a means of diagnosing or measuring progression of AD or any other neurodegenerative disease is an inherently attractive one for the reasons outlined above, and the studies discussed here provide much to be optimistic about. Perhaps one of the main advantages of using the retina is the ability to noninvasively look directly at the nervous system. Much of the evidence discussed above shows that changes in the retina occur later than in the brain. The single transgenic Tg2576 mouse model has been shown to develop A*β* plaques in the brain at 9 months [86], while similar changes occur in the retina at 14 months [109, 111]. There is a similar pattern when looking at double transgenic mouse models with the Tg2576 × Tg1 model showing increased levels of A*β*40 and A*β*42 in the brain by 3-4 months [99] while not being raised in the retina of 7.8-month-old animals [112]. 

However, a recent study looking at double transgenic mice has shown very clearly that A*β* plaques appear earlier in the retina than in the brain by examining the same animals over time [70] and, further, that they can be imaged noninvasively at this early stage. This finding raises the profile of the retina as a potential source of an earlier diagnosis in AD although it remains to be seen whether this finding is replicated in other studies and in human subjects. As discussed earlier, the role of zinc in the formation of A*β* plaques appears to be significant and the fact that the retina is a particularly zinc-rich tissue [134] bodes well for any plaque pathology being detectable relatively early in the disease. 

The rationale for investigating the retina is that, as an extension of the CNS, it is reasonable to expect to find similar changes as occur in the brain. A side effect of this research, however, has been the finding that A*β* deposition occurs in the lenses of AD subjects [61] and has been found in lenses and corneas of single and double transgenic animal models [111]. A*β* deposition and hyperphosphorylated tau have also been detected in the lenses and corneas of triple transgenic mice [81] raising the slightly unexpected but equally welcome possibility that an ocular biomarker for AD may exist that is not connected with the CNS.

Although great progress has been made in this area, there remain significant questions. Firstly, A*β* plaques have only been detected in human AD subjects in one study [70], and it is this same study that provides the only evidence that retinal pathology precedes brain pathology; both findings that need to be corroborated. Secondly, there appears to be significant crossover of AD with other causes of neurodegeneration. Glaucoma, for example, is a neurodegenerative disease that results in loss of RGCs and manifests with RNFL thinning and visual field defects (i.e,. similar findings as those described for AD patients), that have been reported as having a higher incidence in AD [56]. It is possible that the changes thus far reported in the eyes of AD patients and animal models are not as specific for AD as we might hope. 

Thirdly, putting aside the question as to whether changes in the eye precede those of the brain, it is suggested that cognitive deficits of AD may come before detectable amyloid pathology in the brain [99] meaning that a holy grail of detecting AD before its symptoms manifest (implying significant loss of neurons) by detecting amyloid plaques may not be possible. 

Nevertheless, as an absolute minimum, the ability to image the retina (and rest of the eye) noninvasively and relatively cheaply and quickly cannot but massively aid in assessing possible treatment effects of anti-AD, therapies as well as improve our knowledge of the underlying mechanisms of this and other forms of neurodegeneration. In common with AD there is much evidence linking zinc dyshomeostasis with the onset of AMD, the leading cause of blindness in the developed world, with large amounts of zinc found in drusen [133]. An apparent contradiction is that zinc supplements (presumably in their antioxidant role) have been shown to be beneficial in the nonneovascular type of AMD (so called dry AMD) [135]. This is putatively explained in a review by Nan et al. [136] by the fact that, as mentioned above, zinc causes aggregation of CFH. The high concentration of zinc found in drusen leads to a localised aggregation of CFH causing the sustained inflammatory response necessary for initiation of the disease. Later in the disease, the tissue surrounding the zinc-rich drusen is relatively zinc depleted and hence supplementation is beneficial. This is a typical example of the commonalities between different neurodegenerative diseases and highlights how research into one area is likely to benefit in others.

Overall, the research looking at manifestations of AD in the eyes of animal models is notable by its paucity and it is difficult at this early stage to draw any firm conclusions other than that this is an extremely promising area of investigation and certainly warrants further research.

## Figures and Tables

**Table 1:** Hypotheses implicated in the development of Alzheimer's disease.

Mechanisms implicated in AD	Pathophysiology	References
Amyloid hypothesis	Aggregation of A*β* peptides produces oligomers resulting in neurotoxicity and neuronal loss	[13–16]
Tau hypothesis	Hyperphosphorylation of tau proteins causes misfolding of microtubules, which leads to formation of NFT and disruption of the neuronal cytoskeleton	[17, 18]
Cholinergic hypothesis	Loss of cholinergic neurotransmission in the cerebral cortex. Oldest hypothesis on which current available treatments are based on	[12]
Glutamatergic hypothesis	This hypothesis links AD to neuronal damage caused by overactivation of N-methyl-d-aspartate (NMDA) receptors by glutamate. It is suggested that low activation of NMDA receptor is essential for learning and memory	[10]
Oxidative Stress hypothesis	In AD brains, A*β* generates reactive oxygen and nitrogen species which react with other molecules to form free radicals causing molecular and cellular damage Oxidative damage is thought to be early in AD progression because of its link with plaques and NFT.Oxidative stress by A*β* has been shown to be mediated by metal ions	[7, 9, 19]
Chronic Inflammation hypothesis	During AD, cytokines, reactive oxygen species, complement proteins, and prostaglandins are produced to cause chronic inflammation	[11]

**Table 2:** Abnormalities detected in the visual pathway of AD patients.

Visual changes	Manifestations	References
Visual acuity	Visual acuity changes reported in AD patients	[50, 51]
Contrast sensitivity	Several studies have reported changes in contrast sensitivity in Alzheimer's patients compared to controls	[48, 52, 53]
Colour vision	Although controversial, many studies have demonstrated colour vision deficiencies in AD patients, others reporting prevalence to be high	[54–56]
Visual field	There are reports to suggest that AD patients exhibit visual field defects	[57, 58]
Pupillary function	AD patients have exhibited atypical pupil response to cholinergic agonists and antagonists	[59, 60]
Lens	AD patients appear to be predisposed to a particular type of cataract (equatorial supranuclear)	[61]
Retina	AD patients have been shown to have a specific pattern of RNFL thinning, perhaps related to the severity of AD. Also shown to have decreased retinal blood flowChanges in retinal function using electrophysiological tests (PERGs and VEPs) have been shownA*β* plaques have been demonstrated in the retinas of AD patients	[62–70]

**Table 3:** Retinal changes documented in AD transgenic animal models.

Type	Mutations	Age	A*β* in retina	A*β* plaques	A*β* in retinal vasculature	APP immunoreactivity	Tau deposits	Neuroinflammation	Neuronal cell loss	References
Single	APP_swe_ double K595N/M596L	14 months	GCL, IPL, INL, OPL, ONL	Yes	Retinal capillaries	GCL, INL	GCL, IPL, INL, OPL, ONL	Detected in all layers of retina	Yes	[105]

Single	APP_swe_ double K595N/M596L	14 months	GCL	No	n/a	GCL, INL	n/a	n/a	n/a	[106]

Single	APP_swe_ double K595N/M596L	24 months	GCL, INL, ONL	No	n/a	Yes—layers not specified	n/a	n/a	n/a	[109]

Single	PS1 knock-in	12, 15 and 30 months	Not detected	No	n/a	Not detected	n/a	n/a	n/a	[109]

Single	Human P301S tau	2–5 months	n/a	Yes	n/a	n/a	RNFL, GCL	n/a	No	[110]

Double	APP_swe_/PS1_M146L_	7.8 months	Virtually absent	Yes	Not detected	GCL, INL	n/a	GCL (less than at 27 months)	GCL (less than at 27 months)	[107]

Double	APP_swe_/PS1_M146L_	27 months	RNFL, GCL	Yes	Retinal and choroidal	GCL, IPL, INL, OPL, OS, RPE	n/a	GCL, IPL, OPL	GCL	[107]

Double	APP_swe_/PS1_ΔE9_	10.5 months	RNFL	Yes	Choroidal only	GCL	n/a	GCL, IPL	GCL	[107]

Double	APP_swe_/PS1_ΔE9_	9 months	GCL, IPL, INL, OPL	No	n/a	IPL, OPL	n/a	n/a	n/a	[106]

Double	APP_swe_/PS1_ΔE9_	12–21 months	IPL, OPL	IPL, OPL	n/a	n/a	n/a	n/a	No	[108]

Double	APP_swe_/PS1_ΔE9_	24 months	GCL, INL, ONL	No	n/a	Yes—layers not specified	n/a	n/a	n/a	[109]

Double	APP_swe_/PS1_ΔE9_	2.5–17 months	RNFL, GCL, IPL, INL, OPL, OS	RNFL, GCL, IPL, INL, OPL, OS	n/a	n/a	n/a	n/a	n/a	[69]

Triple	APP_swe_/PS1/P301L	18 months	n/a	n/a	n/a	n/a	n/a	n/a	Yes	[45]

Triple	APP_swe_/PS1/P301L	18 months	GCL, OS	GCL, OS	n/a	n/a	GCL, IPL, INL, OPL, ONL, OS	n/a	n/a	[111]

*RNFL:* retinal nerve fibre layer,* GCL: *ganglion cell layer, *IPL:* inner plexiform layer, *INL:* inner nuclear layer, *OPL:* outer plexiform layer, *ONL:* outer nuclear layer, *OS:* photoreceptor outer segments.
